# BDNF Met66 modulates the cumulative effect of psychosocial childhood adversities on major depression in adolescents

**DOI:** 10.1002/brb3.220

**Published:** 2014-02-09

**Authors:** Carlos S Cruz-Fuentes, Corina Benjet, Gabriela A Martínez-Levy, Amado Pérez-Molina, Magdalena Briones-Velasco, Jesús Suárez-González

**Affiliations:** 1 Psychiatric Genetics Department, Clinical Research Branch, Instituto Nacional de Psiquiatría “Ramón de la Fuente Muñíz”Mexico City, México; 2 Epidemiology and Psychosocial Research Division, Instituto Nacional de Psiquiatría “Ramón de la Fuente Muñíz”Mexico City, México

**Keywords:** Adolescence, brain-derived neurotrophic factor gene, childhood adversities, major depression, Mexican, serotonin transporter gene

## Abstract

**Background:**

The interplay among lifetime adversities and the genetic background has been previously examined on a variety of measures of depression; however, only few studies have focused on major depression disorder (MDD) in adolescence.

**Methods:**

Using clinical data and DNA samples from mouthwash gathered from an epidemiological study on the prevalence of mental disorders in youths between 12 and 17 years old, we tested the statistical interaction between a set of psychosocial adversities experienced during childhood (CAs) with two common polymorphisms in the brain-derived neurotrophic factor (BDNF) (Val66Met) and SLC6A4 (L/S) genes on the probability of suffering MDD in adolescence.

**Results:**

Genotype or allele frequencies for both polymorphisms were similar between groups of comparison (MDD *N* = 246; controls *N* = 270). The CAs factors: Abuse, neglect, and family dysfunctions; parental maladjustment, parental death, and to have experienced a life-threatening physical illness were predictors of clinical depression in adolescents. Remarkably, the cumulative number of psychosocial adversities was distinctly associated with an increase in the prevalence of depression but only in those Val/Val BDNF individuals; while the possession of at least a copy of the BDNF Met allele (i.e., Met +) was statistically linked with a “refractory” or resilient phenotype to the noticeable influence of CAs.

**Conclusion:**

Liability or resilience to develop MDD in adolescence is dependent of a complex interplay between particular environmental exposures and a set of *plasticity* genes including BDNF. A better understanding of these factors is important for developing better prevention and early intervention measures.

## Introduction

Major depressive disorder (MDD) manifested in adolescence is common, recurrent, and often perpetuated into adulthood (Fombonne et al. 2001). MDD in adolescents frequently occurs in comorbidity with other psychiatric disorders and is an important contributor to increased risk of suicide, substance abuse, and behavioral problems (Harrington et al. 1994; Yorbik et al. 2004). Moreover, it disrupts occupational, social, emotional, and physical health and is frequently associated with poor psychosocial and academic outcome carrying considerable stigma (Fletcher 2008; Thapar et al. 2012).

The etiology of MDD is considered complex and multifactorial, involving a purported interplay of multiple environmental and genetic factors (Kendler et al. 2013). In this regard, a miscellaneous set of distressful psychosocial events experienced early in life (e.g., maltreatment, neglect, abuse) has been consistently associated with an increased risk to manifest major depression (Kendler et al. 2000; Jaffee et al. 2002).

On the other hand among the numerous candidate genes evaluated with regard to MDD, those coding for the brain-derived neurotrophic factor (BDNF) and the serotonin transporter (SERT; 5HTT) have been particularly appealing for genetic association studies. Both molecules participate in cellular signaling systems that regulate the development and plasticity of neural circuits involved in depression and anxiety (Martinowich and Lu 2008; Castrén and Rantamäki 2010); in addition a variety of cellular and molecular reciprocal interactions between BDNF with the serotonin (5HT) neural system exists (Martinowich and Lu 2008).

In particular, two common genetic variants have been recurrently tested: the 44 pair base insertion/deletion polymorphism in the promoter region of the SLC6A4 gene (aka 5HTT-LPR), yielding long (L) or short (S) alleles; and the single-nucleotide polymorphism (A/G: rs6265) which predicts the substitution of valine to methionine at codon 66 in the prodomain of BDNF gene (Val66Met). Several studies have documented the interaction analyses between specific lifetime adversities and these specific candidate genes (analyzed either independently or simultaneously) on a variety of measures of depression (Kaufman et al. 2006; Kim et al. 2007; Wichers et al. 2008; Nederhof et al. 2010; Carver et al. 2011; Grabe et al. 2012). However, only few studies focused in major depression disorder during adolescence. Employing clinical data and biological samples for genetic analysis gathered from the Mexican Adolescent Mental Health Survey, we tested the hypothesis that the risk for developing clinical depression would be dependent on the individual and/or cumulative effect of psychosocial adversity factors but moderated by genetic variants; this outcome should be particularly evident for those individuals bearing the BDNF Met allele (i.e., Met/Met and Met/Val) and/or homozygous for the SLC6A4 short allele.

## Methods

Initiated in 2005 under the auspices of the World Health Organization, the Mexican Adolescent Mental Health Survey (MAMHS) was designed to generate estimations of the prevalence of 20 major psychiatric disorders experienced during adolescence (for specific details of the experimental design see Benjet et al. 2009a,b2009b). Participants were intended to be representative of the approximately 2 million youths between 12 and 17 years old, inhabitants of the metropolitan area of Mexico City.

Briefly, 3005 individuals were interviewed face-to-face in their homes by lay personnel trained in the use of the computer-assisted World Mental Health Composite International Diagnostic Interview for Adolescents (WMH-CIDI-A; Merikangas et al. 2009). This comprehensive, fully structured interview was designed to assess the most prevalent psychiatric disorders according to the definitions and criteria of ICD-10 and Diagnostic Statistical Manual IV (DSM-IV). This study focused on MDD: the lifetime diagnosis of major depression was attained from the report on depressive symptoms and based in DSM-IV criteria (American Psychiatric Association 1994). The clinical validity of CIDI in relation to standardized clinical assessments has been discussed elsewhere (Haro et al. 2006). The clinical algorithm included in WMH-CIDI-A is able to differentiate between those cases whose depression is related with other disorders; therefore, in this study we apply the diagnostic algorithm with a hierarchical rule, stating that if a disorder is better explained by another mental disorder, that “other” disorder is given hierarchy over the disorder of interest. Post-hoc analysis included also the nonhierarchical criteria in order to allow assessment of psychiatric comorbidity. In this study, the lifetime prevalence of MDD was in agreement with other published studies (Waraich et al. 2004; Essau and Chang 2009; Ferrari et al. 2013). Two hundred and forty-six adolescents that met these clinical criteria were also able to donate a mouthwash sample for genetic analyses.

The group of noncases (i.e., controls) for genetic typing was obtained from the same database after excluding all those subjects who met criteria for any other psychiatric disorder. From this set of 1261 subjects who did not classify for any psychiatric diagnosis, 875 agreed to donate a mouthwash. For this study 270 samples were analyzed; they were not different with regards to the main sociodemographic variables: average age, female: male ratio, or percentage of subjects who met criteria for economic adversity from the original set from where they were chosen (data not shown).

### Ethical considerations

This study was conducted in accordance with the ethical principles of the Declaration of Helsinki and was approved by the Ethics and Scientific Committees of the National Institute of Psychiatry “Ramón de la Fuente Muñíz” (INPRFM) in Mexico City. Interviewers gave a verbal and written explanation of the study and obtained informed consent from the parent or legal guardian and the assent of the adolescent.

#### Childhood psychosocial adversities

In addition to the psychiatric survey, information about psychosocial adverse risk factors experienced the previous years was collected. A set of 12 childhood adversities (CAs) experienced during childhood was analyzed. They were evaluated from the childhood and posttraumatic stress disorder sections of the WMH-CIDI-A as described elsewhere (Benjet et al. 2011). The selection and scoring of these measures are the same as that created for the World Mental Health Survey (Greif Green et al. 2010).

All adversities were considered chronic because of reporting of multiple accounts or continued occurrence. A factor analysis showed three meaningful components in this subsample (data available on request), similar to results obtained in the whole sample (Benjet et al. 2009b): The first factor was comprised of six CAs regarding family dysfunction, abuse and neglect, and parental maladjustment variables; criminality and substance abuse described the second factor; finally, a third factor included the report of parental divorce associated with extreme family economic adversity. On the other hand, to have experienced the death of a parent was relatively independent of being exposed to other CAs, while having a life-threatening physical illness in childhood was also moderately independent of other adversities, although it loaded with family dysfunction adversities.

### Molecular analyses

High-molecular-weight DNA (≅23 Kb) was extracted from mouthwash samples using the Puregene DNA purification Kit (Qiagen™, Hilden, Germany). A detailed analysis on the quality of the nucleic acids obtained and the efficiency of PCR amplification achieved is available on request.

Genotyping of SLC6A4 promoter VNTR (5HTT-LRP) was determined by agarose gel size fractionation as we have previously reported (Camarena et al. 2001). Gels were read in a blind fashion by two different evaluators obtaining a 100% concordance. Alleles were designated according to their relative size: S (14 repeats), L (16 repeats), with no other rare alleles detected in this sampling. Genotyping of rs6265 in the BDNF gene (i.e., Val 66 Met) was performed using the TaqMan 5′ exonuclease assay C_11592758_10 from Applied Biosystems™ (Foster City, CA).

In order to ensure the correct identification of genotypes, 10% of all samples were randomly reanalyzed obtaining a 100% of concordance.

As we recently reported (Velazquez-Aragón et al. 2012), a panel of 10 ancestry informative markers that can distinguish between Amerindian and European ancestry in Mexican populations (*δ* > 0.44) was genotyped in a random group of 200 samples. The STRUCTURE software was used to test stratification within samples. An admixture model was employed using the following parameters: 1 × 10^6^ of burn-in period, 1 × 10^6^ repetitions, and *K* = 2. These markers have been validated in previous case–control studies in the Mexican population. Values of *α* > 1 obtained indicated that there was no population substructure in our sample.

For the 44 bp SCL64 polymorphism, analyses were initially performed including the three genotypes. A post-hoc analysis combined genotypes L/L and S/L in order to increase statistical power. In the case of BDNF, analyses were performed collapsing Met/Met and Met/Val individuals in a single category (i.e., Met +), as Met homozygous individuals were infrequent (i.e., 2% of the whole sample).

### Statistical analysis

The selection of samples for this study was obtained from the epidemiological database (stored in the SAS/STAT format), which contains the clinical algorithms for depression and noncases as described above. For continuous variables (e.g., age) an *F* statistic was employed as test for statistical significance in a one-way ANOVA, otherwise comparison of categorical variables (e.g., gender or genotypes) were made using *χ*^2^ tests. Hardy–Weinberg equilibrium (HWE) was tested with the online test of deviation of HWE program (Rodriguez et al. 2009). A logistic regression analysis was carried out to examine the effects of main covariates and effect modification (i.e., interaction) for depression as the output variable. The putative association of genetic variants and environmental adversities were analyzed with a multinomial logistic regression analysis (SPSS v.20, Chicago, IL).

## Results

Table 1 shows the descriptive statistics of the demographic variables analyzed in the sample: cases and controls were similar, with exception of a slight but statistical significant increment in average age in those affected individuals.

**Table 1:** Comparison of sociodemographic variables and BDNF and SLC6A4 genotype frequencies in cases and controls.

		MDD	Controls	Statistics
Age		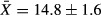	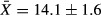	*t* = −4.7, gl = 457, *P* = 0.00
Gender	Males	32.9% (70)	59.3% (146)	*χ*^2^ = 2.6, gl = 1, *P* = 0.14
	Females	61.3% (143)	40.7% (100)	
Economic adversity		26.8% (57)	21.5% (53)	*χ*^2^ = 1.7, gl = 1, *P* = 0.19
SLC6A4	LL	11.7% (25)	14.2% (35)	*χ*^2^ = 3.4, gl = 2, *P* = 0.18
	LS	42.7% (91)	46.8% (115)	
	SS	45.5% (97)	39% (96)	
BDNF	Met/Met	1.9% (4)	2% (5)	*χ*^2^ = 0.01, gl = 2, *P* = 0.99
	Met/Val	21.6% (46)	21.5% (53)	
	Val/Val	76.5% (163)	76.4% (188)	

BDNF, brain-derived neurotrophic factor; MDD, major depression disorder.

### Analysis of *BDNF and SLC6A4* with MDD

Table 2 also shows the observed genotype frequencies for the SLC6A4 promoter VNTR and the BDNF Val66Met polymorphisms in cases and controls; no deviation from HWE was detected in both cases: SLC6A4, *χ*^2^ = 0.4, *P* > 0.05; BDNF, *χ*^2^ = 0.3, *P* > 0.05. Moreover, no significant genotype or allele differences were observed between groups of comparison. However, when hierarchical diagnostic criteria for depression was used as the output variable, the *SS SLC6A4* genotype (but not *BDNF*) was a predictor of major depression (Wald's statistic 5.8, *P* = 0.016, CI = 1.1–2.7). No significant gene × gene interaction was detected (Wald 0.54, *P* = 0.461).

### Analysis of CAs

With exception of parent divorce and economic adversity, most of the 12 individual CAs were independent predictors of depression as analyzed by logistic regression (Wald statistic range 13–110, *P* < 0.001; individual data not shown).

The psychosocial adversity composite factors: Abuse, neglect, and family dysfunctions (Wald's 88.2, OR 3.6; CI 2.7–4.6; *P* < 0.001); parental maladjustment (Wald's 8.2, OR 1.9; CI 1.2–2.9; *P* < 0.01), parental death (Wald's 6.5, OR 2.0; CI 1.2–3.4; *P* < 0.01), and to have experienced a life-threatening physical illness (Wald's 7.0, OR 1.9; CI 1.2–3.1; *P* < 0.01); were predictors of clinical depression in adolescents. Similar results were observed when data was analyzed by gender, except for the cases of parental maladjustment and parental death where the statistical significance was detected only in female subjects or in male subjects, respectively (Table 2)**.**

**Table 2:** Logistic regression analysis of the influence of childhood adversity factors and candidate genes on the probability of belonging to the MDD category.

Adversities	Males			Females		
	*P*	OR	CI 95%	*P*	OR	CI 95%
Abuse, neglect, and family dysfunction	0.00*	2.9	1.8–4.5	0.00*	3.9	2.7–5.4
Parental maladjustment	0.01*	2.3	1.2–4.5	0.06	1.7	0.9–3.0
Life-threatening childhood physical illness	0.02*	2.3	1.1–4.6	0.03*	2.2	1.1–4.6
Parental death	0.18	1.6	0.8–3.1	0.03*	2.0	1.0–3.8
Parental divorce/economic adversity	0.60	1.1	0.7–1.8	0.98	1.0	0.7–1.4
SCL6A4 SS genotype	0.23	1.4	0.8–2.8	0.37	1.3	0.7–2.3
BDNF Met + genotype	0.13	2.3	0.8–3.5	0.69	1.6	0.6–2.3

BDNF, brain-derived neurotrophic factor; CI, confidence interval; MDD, major depression disorder; OR, odds ratio.

* *P* < 0.05.

**Figure 1:**
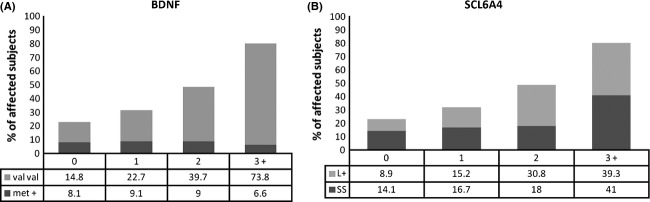
Bars represent the percentage of subjects who met DSMIV criteria for Major depression disorder in relation to the cumulative number of CAs experienced during childhood. The specific percentages of affected individuals relative to a particular genotype (A) BDNF or (B) SLC6A4 are showed as shaded areas or indicated in the corresponding tables. BDNF, brain-derived neurotrophic factor; SCL6A4, serotonin transporter gene.

The cumulative number of psychosocial adversities was clearly associated with an increase in the prevalence of depression (Fig. 1A and B). The logistic regression analysis showed that being exposed to ≥2 CAs during childhood was an important predictor of MDD as compared with those adolescents that reported none or a single childhood adversity (Wald's 44.9, OR 4.5; CI 2.9–6.9; *P* < 0.00). Interestingly, whereas homozygous subjects for the BDNF Val allele displayed an analogous pattern to the whole sample, the possession of at least a copy of the BDNF Met allele (i.e., Met +) was statistically associated with a “refractory” or resilient phenotype to the mounting influence of CAs **(**Fig. 1A). In support of the preceding observation, the *BDNF genotype* × *number of reported CAs* interaction analysis showed a protective effect of the Met allele on the risk for MDD (Wald's 6.5, OR 0.2; CI 0.09–0.7; *P* < 0.02); this effect was only evident in females (Table 3). No significant differences for the interaction of cumulative number of adversities and SLC6A4 were detected (Fig. 1B).

**Table 3:** Interaction analysis by gender between the cumulative number of childhood adversities (CAs) factors and BDNF on the probability of belonging to the MDD category.

	Males			Females		
	*P*	OR	CI 95%	*P*	OR	CI 95%
None or 1 versus ≥2 CAs	0.00*	5.3	2.4–11.4	0.00*	6.0	2.8–12.9
BDNF Met +	0.15	1.8	0.8–2.8	0.27	1.5	0.7–2.9
Non or 1 versus ≥2 CAs × BDNF Met +	0.30	0.5	0.1–2.0	0.01*	0.1	0.02–0.7

BDNF, brain-derived neurotrophic factor; CI, confidence interval; MDD, major depression disorder; OR, odds ratio.

* *P* < 0.05.

## Discussion

In this report, we examined the putative interaction of a set of 12 psychosocial adversities experienced during childhood in relation to the candidate genes BDNF and SLC6A4 on their impact on major depression in adolescence. To our knowledge, this is the first report for a Latin American population. The joint influence of these genes on depression has been frequently examined in regards to a heterogeneous set of “environmental” disadvantageous variables, including maltreatment in childhood (Kaufman et al. 2006), “risky families” (Carver et al. 2011), CAs (Wichers et al. 2008; Aguilera et al. 2009; Grabe et al. 2012), pre/peri gestational difficulties, and family and stressful events during childhood (Nederhof et al. 2010), or threatening events during the last year in the elderly (Kim et al. 2007).

We found as expected, a strong association between most of the CAs studied and the manifestation of clinical depression, either if they were analyzed as independent variables or included as adversity factors. Moreover, there was a clear-cut effect of the cumulative number of CAs on the increase in the prevalence of major depression. On the other hand, when genetic data were analyzed independently of CAs, SLC6A4 (SS genotype) but not BDNF showed a marginal but statistically significant association with the disorder. It is worth noting that the frequently cited drawback of the probability of spurious positive or negative results of case–control genetic association studies as result of population stratification bias was reduced as both groups of comparison were drawn from the same set of individuals. Moreover, cases as well as controls were part of a single admixed population as indicated by the analysis of ancestry markers.

The absence of a main genetic effect for BDNF was not unexpected***;*** for example, genetic association studies related to BDNF Val66Met and mood disorders have frequently produced mixed or negative results as have been showed in two recent meta-analyses (Gratacòs et al. 2007; Verhagen et al. 2010). Moreover, a previous meta-analysis did not detect a significant association between the short allele of the 44-bp SLC6A4 insertion/deletion polymorphism and unipolar depression (Lasky-Su et al. 2005).

Remarkably, a “*refractory*” or resilient phenotype to the mounting influence of CAs in those adolescents bearing the Met 66 allele was noted, which emphasizes the importance of including “environmental and genetic data” in the identification of liability or resilience phenotypes. The “protective” effect of the BDNF Met allele was in the opposite direction to our initial hypothesis, which was based upon experimental observations in humans indicating striking brain anatomical and functional differences among genotypes. For example, as compared with those Val/Val subjects, Met allele carriers showed a reduced hippocampal gray matter volume (Pezawas et al. 2004; Szeszko et al. 2005; Bueller et al. 2006), a less efficient verbal episodic memory, and an abnormal hippocampal activation on performing a working memory task response (Egan et al. 2003) or a decreased probability of using a hippocampus-dependent spatial strategy (Banner et al. 2011). Reasons for this discrepancy could be attributed to differences in study designs; for example, while most studies have employed measures of depressive symptomatology or negative affect as the output variable, clinical diagnosis of depression has rarely been evaluated. However, it is worth noting that the Val allele was the risk marker associated to depressive symptoms and/or anxiety-related traits in some of these reports (Sen et al. 2003; Lang et al. 2005; Duncan et al. 2009). Other potential confounder might be the human developmental stage investigated in the present study, as we focused particularly in adverse experiences in childhood on psychiatric disorders in adolescence. Finally, divergent outcomes have been reported that might be contingent of the specific variables analyzed, including: the type and/or time of stressor (maltreatment vs. threatening events; early vs. late adversities); the clinical manifestation of depression (single episode vs. recurring or chronic course); or the brain areas studied (hippocampus vs. amygdala vs. ventral tegmental area).

Some selected work can illustrate this point. The first example was provided by Krishnan et al. (2007), who showed a striking behavioral difference in genetically engineered mice bearing the human Met/Met genotype. These animals (as opposed to the homozygous Val/Val mice) were unsusceptible to an otherwise important reduction in social interaction after being exposed to a social defeat paradigm. Moreover, the behavioral resilience was also related to an important reduction in the BDNF protein levels in the *nucleus accumbens*. A second example is provided by Casey et al. (2010), who reported differential effects of the BDNF allelic variation on the brain morphometry of children exposed to an early postnatal adversity condition. Thus, while some studies show a reduction in the hippocampal volume of healthy adult carriers of the Met allele in relation to Val/Val subjects (Pezawas et al. 2004; Szeszko et al. 2005; Bueller et al. 2006); these researchers showed that relative to controls, the volume of this limbic structure was diminished in Val homozygous children that were institutionalized in orphanages within their first year of life; whereas the Met carriers displayed an increased amygdala volume following this social deprivation.

These and other observations underscore the necessity of abandon simple-minded notions of “good versus bad” alleles and rely on a comprehensive analysis of the numerous variables involved in a particular phenotypic outcome for the proper identification of the neurobiological and/or behavioral effects of a specific BDNF Val66Met allele/genotype.

Notwithstanding our results should be evaluated in the context of several limitations. (1) The diagnosis was based on a composite international interview that has been reported to have an adequate concordance with the clinical assessment; however, these results must be replicated in clinical samples. (2) Mouthwash samples were available for approximately 80% of the total sample interviewed. Nonetheless, main sociodemographic variables (e.g., average age; women: men ratio, % economic adversity) were not different between subjects selected for this analysis from those of the whole set of individuals. (3) As result of the transversal design of our study, we could have missed some cases where the debut of clinical MDD were later in life; so the CAs and genetic influences could be extended to MDD lifetime diagnosis. A longitudinal second-wave of MAMHS is under way.

In summary, BDNF and SLC6A4 should be conceptualized as members of a set of “plasticity” genes that modulate the individual susceptibility to develop MDD from particular environmental exposures. Even though considerable advances have been made in our knowledge of early-onset depression, further research is needed in understanding the pathogenesis of childhood mood disorders. Toward this goal, studies aimed at elucidating mechanisms and interrelationships among the different domains of risk factors are needed.
